# Automated Fovea Detection in Spectral Domain Optical Coherence Tomography Scans of Exudative Macular Disease

**DOI:** 10.1155/2016/7468953

**Published:** 2016-08-31

**Authors:** Jing Wu, Sebastian M. Waldstein, Alessio Montuoro, Bianca S. Gerendas, Georg Langs, Ursula Schmidt-Erfurth

**Affiliations:** ^1^Christian Doppler Laboratory for Ophthalmic Image Analysis (OPTIMA), Vienna Reading Center, Department of Ophthalmology, Medical University of Vienna, Vienna, Austria; ^2^Christian Doppler Laboratory for Ophthalmic Image Analysis (OPTIMA), Computational Imaging Research Lab, Department of Biomedical Imaging and Image-Guided Therapy, Medical University of Vienna, Vienna, Austria

## Abstract

In macular spectral domain optical coherence tomography (SD-OCT) volumes, detection of the foveal center is required for accurate and reproducible follow-up studies, structure function correlation, and measurement grid positioning. However, disease can cause severe obscuring or deformation of the fovea, thus presenting a major challenge in automated detection. We propose a fully automated fovea detection algorithm to extract the fovea position in SD-OCT volumes of eyes with exudative maculopathy. The fovea is classified into 3 main appearances to both specify the detection algorithm used and reduce computational complexity. Based on foveal type classification, the fovea position is computed based on retinal nerve fiber layer thickness. Mean absolute distance between system and clinical expert annotated fovea positions from a dataset comprised of 240 SD-OCT volumes was 162.3 *µ*m in cystoid macular edema and 262 *µ*m in nAMD. The presented method has cross-vendor functionality, while demonstrating accurate and reliable performance close to typical expert interobserver agreement. The automatically detected fovea positions may be used as landmarks for intra- and cross-patient registration and to create a joint reference frame for extraction of spatiotemporal features in “big data.” Furthermore, reliable analyses of retinal thickness, as well as retinal structure function correlation, may be facilitated.

## 1. Introduction

Spectral domain optical coherence tomography (SD-OCT) is a noninvasive modality for acquiring high resolution, 3D cross-sectional images of the retina, and is today the most important ancillary test for the diagnosis and management of macular diseases [1]. Usually, serial OCT acquisitions are compared over time to determine disease progression and/or treatment response. However, as a result of acquisitions at multiple time-points, motion or imaging related registration errors commonly occur, that is, scans of the same eye at different time-points may be aligned incorrectly. Such registration artefacts may have a severe effect on the ability to perform accurate and reproducible analysis of subtle changes over time [2–5]. This problem can be overcome by computationally aligning sequential OCT scans using automated registration [2], or by labour-intensive manual alignment of the OCT scans or measurement grids.

In both cases, the fundamental requirement for registration of OCT volumes is the use of adequate anatomical landmarks. Further to the retinal vasculature, which has been used previously to register OCT volumes [2], the fovea centralis is a particularly important registration landmark. For example, correct identification of the foveal position is key for automated retinal thickness assessment using fovea-centered measurement grids such as the early treatment diabetic retinopathy study (ETDRS) grid and rotational alignment of the measurement grid in circumpapillary retinal nerve fiber layer measurements for glaucoma and multiple sclerosis. Due to its relevance for visual acuity, knowledge of the exact foveal position is also critical for studies of structure–function correlation [6]. However, given the immensely large scale of imaging data both in modern clinical practice and research, fully automated analysis methods are required for efficient OCT analysis.

To our knowledge, computational detection of the fovea in SD-OCT has been limited to healthy or dry-AMD cases [7–13]. Thus, the major challenge of this work is to develop a detection method for the fovea in SD-OCT scans of exudative macular disease where the retina has been heavily deformed by the presence of fluid, severely altering the retinal anatomy.

In this article, we present a fully automated fovea detection method that is capable of accurately and reproducibly identifying the position of the fovea in cross-vendor longitudinal OCT scans of both normal and patients suffering from exudative macular disease, that is, cystoid macular edema secondary to retinal vein occlusion (RVO) and neovascular age-related macular degeneration (nAMD). In our method, we consider specific disease morphology to account for the differences between disease types. Our goal is to demonstrate the accuracy of the presented fully automated detection system and thus the systems' feasibility for detection of the foveal position in “big data.”

## 2. Methods

### 2.1. Imaging Data

For this study, 704 clinical SD-OCT imaging datasets from the Vienna Reading Center's (VRC) image database were used. 494 scans were selected from multicenter trials evaluating ranibizumab for central or branch RVO (clinicaltrials.gov identifiers, NCT01535261 and NCT01599650), and 210 scans from a multicenter trial evaluating ranibizumab for nAMD (clinicaltrials.gov identifier, NCT01780935). The study was conducted in compliance with the tenets set forth in the Declaration of Helsinki. The randomized clinical trials from which the scans were obtained were approved by the institutional review board of each participating center. All patients gave written consent for participation in the respective trial and all data was appropriately anonymized.

For method development, distinct training and testing image datasets were constructed. The training set, used to optimize the detection algorithms, consisted of 180 scans, divided into three equally sized groups representing branch RVO, central RVO, and nAMD, randomly selected from the baseline time-point where disease is most prevalent. The unseen testing set (used to validate the final algorithms) was comprised of 240 scans divided into the disease groups branch RVO (42 Heidelberg Spectralis, 33 Zeiss Cirrus, 5 Topcon), central RVO (53 Heidelberg Spectralis, 23 Zeiss Cirrus, 4 Topcon), and neovascular AMD (48 Heidelberg Spectralis, 32 Zeiss Cirrus), comprised of 80 scans each (all distinct from the training set), randomly selected to be inclusive of various acquisition time-points. In both the training and testing datasets, each scan was acquired from a distinct patient.

For all 420 scans, to provide an objective and standardized ground truth, the position of the fovea was manually annotated by expertly trained graders from the VRC using validated custom software [14]. The diagnostic criteria for the foveal center included (1) minimization of the retinal nerve fiber layer (RNFL) thickness; (2) presence of a foveal depression; (3) focal elongation of the photoreceptor outer segment signal, as described previously [15].

Inclusion criteria for dataset construction included acquisition from multiple devices (Zeiss Cirrus, Heidelberg Spectralis, and Topcon 3D OCT 2000) as well as baseline and nonbaseline time-points. Each OCT volume has average physical dimensions of 6 × 6 × 2 mm^3^ (*X*, *Y*, *Z*) with slice thickness ranging from 11.72 *μ*m to 122.2 *μ*m. Dependent on device, this physical dimension may equate to 200 × 200 × 1024 (Zeiss Cirrus), 256 × 256 × 885 (Topcon 3D OCT 2000), 512 × 128 × 885 (Topcon 3D OCT 2000), 512 × 128 × 1024 (Zeiss Cirrus), or 512 × 49 × 496 (Heidelberg Spectralis) pixels.

### 2.2. Fovea Detection Preprocessing

**Figure 1:**
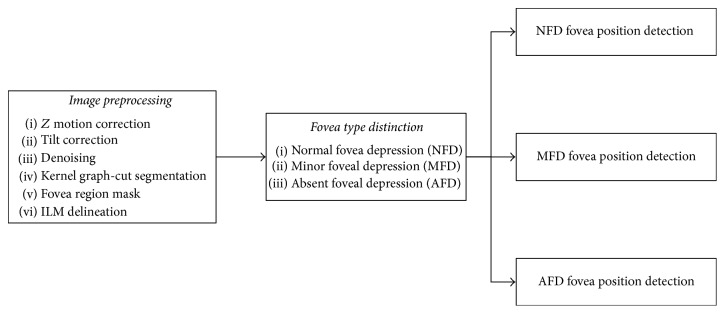
Flow diagram showing the major stages of the fovea detection algorithm. The three major components are comprised of image preprocessing, fovea type distinction, and fovea type based fovea position detection.

The flow diagram in Figure 1 illustrates the three major stages comprising the automated fovea detection algorithm presented here, that is, image preprocessing, fovea type distinction, and fovea type based fovea position detection, which are discussed in detail below.

**Figure 2:**
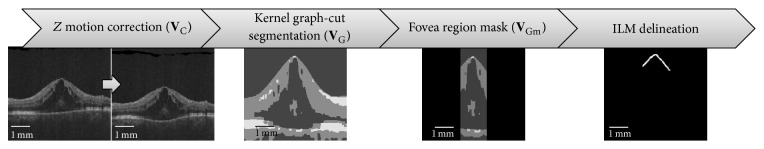
Flow diagram showing overview of fovea detection preprocessing stage. From left to right, the stages, *Z* motion correction, Kernel graph-cut segmentation, fovea region masking, and finally ILM delineation, are illustrated. The delineated ILM surface in the far right image is illustrated as a white curve.

The preprocessing stage is shown in Figure 2. Firstly, motion correction in the *Z* plane is performed on the entire image volume using the local-symmetry estimation method described in [16] to remove motion artefacts caused by microsaccadic eye movement. This can be seen in Figure 2 in the left image showing the retina orientation flattened. Secondly, tilt correction, also described in [16], is performed in the B-scan plane reducing the horizontal tilt of the retina. The resulting motion corrected volume is denoted as **V**_C_.

Thirdly, denoising is performed on **V**_C_ to reduce speckle noise using the block matching collaborative filtering approach described in [17], giving **V**_NF_.

On **V**_NF_, a kernel graph-cut segmentation algorithm [18] is applied to delineate the inner limiting membrane (ILM), as well as intraretinal cystoid fluid (IRF) regions, resulting in the segmented volume **V**_G_. Finally, computational complexity is reduced by masking **V**_G_. A 2D elliptical mask **M**, of size **M**_*x*_ = *X*/2 and **M**_*y*_ = *Y*/2, is constructed in the* XY* plane centered at a statistically generalised mean fovea position, where *X* and *Y* are *X* and *Y* image dimension sizes. This center point has been computed by averaging manually annotated fovea positions from the training dataset, annotated by expert graders at the VRC, and was found to be within *x* = 80 *μ*m and *y* = 140 *μ*m of the scan center, based on their relative distances from the respective scan centers. As a result, the scan center is used, with all image information outside the mask area removed from the *XY* plane which is then propagated into 3D, resulting in a cylindrical region containing the fovea, **V**_Gm_. This cylindrical region is exemplified in the third image of Figure 2, showing the masked B-scan.

After this preprocessing stage, ILM delineation is performed again on **V**_Gm_ to extract the cropped ILM surface for fovea appearance analysis.

### 2.3. Appearance Based Fovea Detection

#### 2.3.1. Fovea Appearance Classification

**Figure 3:**
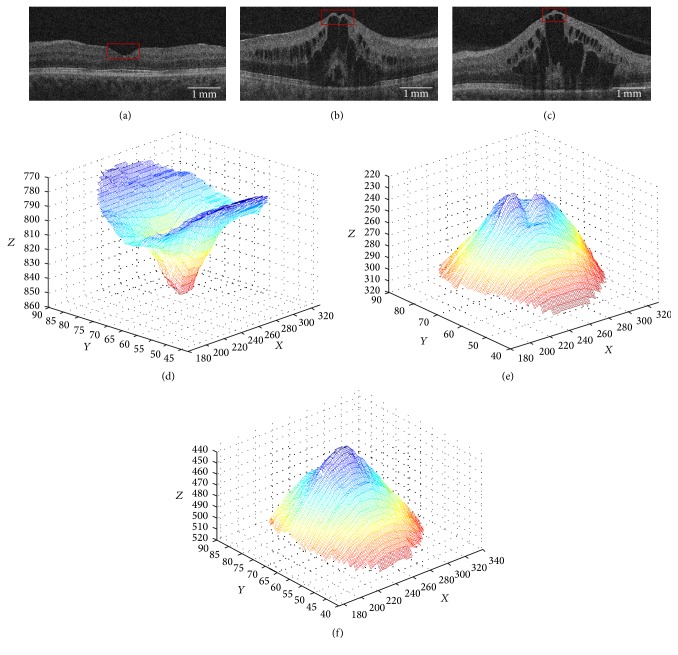
Exemplar SD-OCT B-scan fovea appearances (fovea region outlined in red) (a) normal, (b) minor depression, and (c) absent depression. (d–f) Respective fovea appearances from region outlined in red (a–c) visualized as a 3D mesh.

Given the distinct appearance of the fovea between healthy and diseased, as well as disease-specific variability, different methods for fovea position detection are required. In exudative maculopathy, pathologies that affect the fovea may be categorised into intraretinal cystoid fluid (IRF) resulting in foveal edema, IRF resulting asymmetrical foveal edema, subretinal fluid (SRF), and pigment epithelial detachment (PED). Thus, to further simplify the detection problem, we can generalise the fovea appearance into a small finite number of types based on their appearance when normal and pathological. Three main appearances of the fovea caused by the above described pathologic lesions have been reported previously [19] (Figure 3). In the first case (Figure 3(a)), the fovea appears as a depression which we denote as a* normal foveal depression* (NFD). This is the appearance of the fovea when little to no disease is present. Cases with more severe pathology may be categorised into two major appearances, further exemplified in [20], where the fovea has been deformed by pathology such as IRF.

In* minor foveal depression* (MFD, Figure 3(b)), the fovea appears as a depression smaller than in a NFD case that has been raised in *Z* direction by macular edema. Finally, in* absent foveal depression* (AFD, Figure 3(c)), the fovea is not visible as a depression; instead, the ILM appears as a parabola, again elevated in the *Z* dimension by retinal edema below.

*Automated Distinction of Foveal Appearance Types*. Automated computational diagnosis of the NFD type examines the RNFL layer thickness using the clinical description of minimum RNFL thickness to describe the fovea in normal cases. This method has been previously described in detail in [19].

Distinction of the MFD type focusses on the unique morphology of this fovea appearance in the form of a minor depression that has been elevated in* z*-axis primarily due to IRF regions (Figure 3(b)). From the ILM segmentation in **V**_Gm_ computed in the preprocessing stage (Figure 3(e)), maxima examination of this surface section is performed on a B-scan by B-scan basis. For the scan to feature a MFD, the masked surface must be comprised of 2D surface segments treated as a curve function featuring a global maximum and a further local maximum representing the two peaks seen in Figure 3(b). A peak is defined as a data point in the curve that is larger than its two neighbouring data points. Confirmation of maxima presence is performed by extension into the third dimension, where the required maxima configuration must be present across a contiguous distance of 150 *μ*m, given a mean fovea centralis diameter of 1.5 mm. A physical distance is used rather than number of B-scans due to cross-vendor variable slice thickness. This is further exemplified in Figure 3(e) as a mesh representation of an exemplar MFD showing the two computed peaks.

Distinction of the AFD is based on the identification of a global maximum, across a contiguous 150 *μ*m distance, similar in appearance to a conical shape, as seen in Figure 3(f).

#### 2.3.2. Fovea Position Detection

From the fovea appearance classification stage, a given retinal SD-OCT volume is classified as featuring one of the three fovea appearances described in Section 2.3.1. In the fovea position detection stage, appearance specific detection functions have been developed to compute the fovea position, as described in this section.

**Figure 4:**
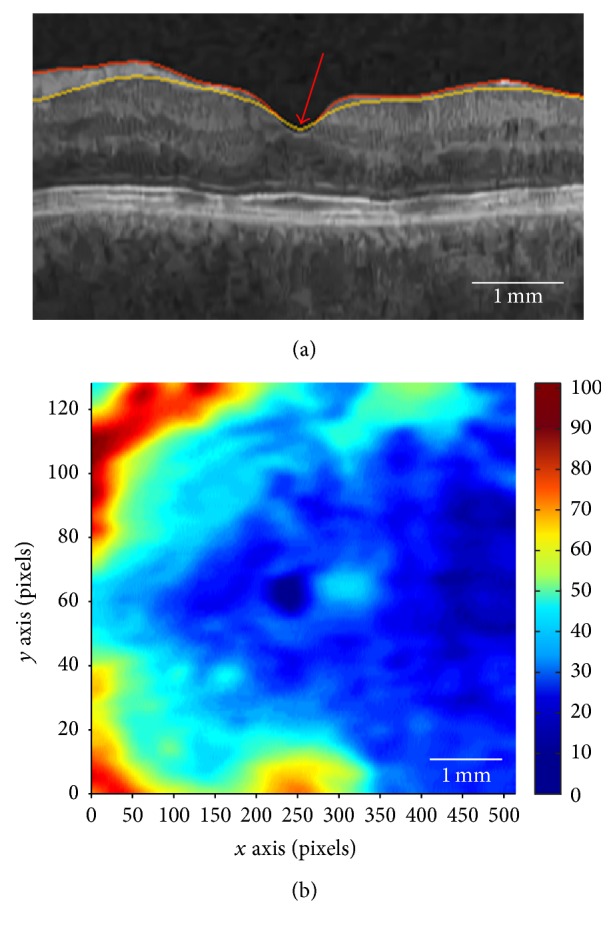
(a) Exemplar ILM (red) and RNFL (yellow) surface segmentations with zero thickness region at the red arrow. (b) RNFL thickness map showing the dark blue zero thickness region in the center. Foveal masking excludes zero thickness regions in the temporal retina.

*NFD Fovea Detection.* In regard to anatomical features, we know that the fovea is the point at which the RNFL thickness is zero. Thus, in the NFD case, we can delineate and extract the two required surfaces (ILM and RNFL) using the graph-cut based retinal surface segmentation algorithm described in [21]. In the NFD detection method, we are only interested in zero thickness in the fovea region, which has been masked and represented as **V**_Gm_. NFD fovea position detection is illustrated in Figure 4.

In the event that multiple zero thickness points are identified, the center of mass is computed and taken as the fovea position. This method is universally applicable to NFD scans.

**Figure 5:**
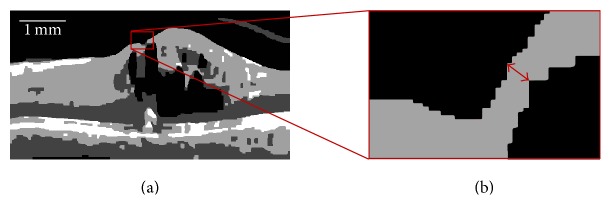
Exemplar B-scan showing MFD (a). Foveal region outlined in red and magnified to show the minimum distance between the ILM surface and IRF (b), used as an indicating feature of fovea position.

*MFD Fovea Detection*. The minimum thickness method for computing fovea position described in the NFD case can no longer be relied upon in the MFD case as this foveal appearance features a retina that has been deformed by pathology such as IRF. As a result, accurate RNFL segmentation is no longer reliable, specifically in the region of interest around the fovea where retinal edema is most prevalent. Thus, to locate the position where the RNFL is thinnest, we compute the distance between the ILM surface and the IRF causing the disruption in the retinal anatomy, as illustrated in Figure 5. Analysis of the manual fovea positions in the training dataset has shown that in all cases where the normal foveal depression was elevated by the presence of IRFs and creating the MFD, the B-scan on which the fovea annotation was performed corresponded to the B-scan where the distance between ILM and IRF boundary was thinnest in all corresponding cases.

However, due to the appearance and morphology of the deformed pathological fovea region, accurately computing the distance may be challenging. Thus, an additional preprocessing step is required, that is, ILM delineation of **V**_NF_ in pathological scans.

Due to the graph-search based retinal surface segmentation algorithm described in [21] performing less adequately in pathological scans as opposed to normal scans, a proprietary ILM segmentation algorithm was developed based on the kernel graph-cut method described in [18]. In this multiregion approach, image partitioning is achieved using kernel mapping of the SD-OCT B-scan. Each B-scan is transformed implicitly by a kernel function in order to apply graph-cut formulation to the problem. In this case, we applied *σ* = 2 to describe the number of regions to segment, as well as a relaxed smoothing constraint to ensure that the ILM surface boundary is delineated as accurately as possible, as opposed to the smoothed surface from [21]. This ensures that the ILM surface used for RNFL thickness measurement in pathological cases is accurate. Vector *p*_ILM_(*x*, *y*) describes this surface.

From **V**_Gm_, given delineated ILM from pathological scan and that the IRF below the fovea is labelled as the lowest intensity region within the retina delineated in **V**_G_, the distance between ILM and IRF is computed to calculate the fovea position.

**Figure 6:**
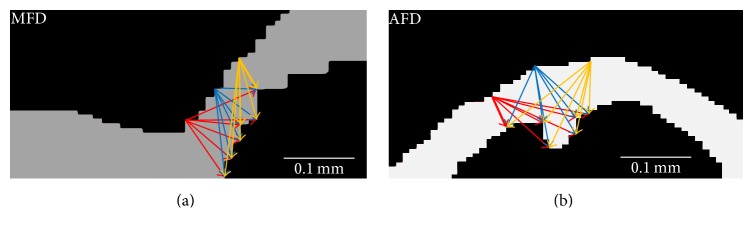
(a) Graphical representation of minimum distance computation between ILM surface (upper) and IRF boundary (lower) in MFD. (b) Exemplar minimum distance computation between ILM and IRF in AFD.

Vector (*p*_IRF_(*x*, *y*)) is computed from the segmented candidate IRF region from within **V**_Gm_ comprised of the boundary points of the region. Furthermore, the minimum distance between ILM (upper surface, Figure 6) and IRF points (lower surface, Figure 6) is computed using pairwise Euclidean distance computation (1) between *p*_ILM_(*x*, *y*) and *p*_IRF_(*x*, *y*) (arrows, Figure 6):

(1) $$\begin{array}{c} d_{ILM\;\;IRF}^{2}=\left(\;p_{ILM}-p_{IRF}\;\right){\left(\;p_{ILM}-p_{IRF}\;\right)}^{\prime }, \end{array}$$

where *d* is the vector of distances between the vector of points *p*_ILM_ (ILM surface) and *p*_IRF_ (IRF surface).

The resulting pairwise distances are sorted in ascending order (ignoring the anatomically impossible zero thickness), choosing the shortest distance (thickest arrows, Figure 6) and the corresponding point from *p*_ILM_(*x*, *y*). However, the volumetric characteristic is also important to consider as the retinal SD-OCT scan is a volume. Thus, similar to the appearance classification phase, the fovea detection algorithm is performed on every B-scan in the masked fovea region in the event multiple B-scans that have identical minimum distances. In this event, the center of mass is computed as the fovea position in the *XY* plane with *Z* position obtained from the ILM segmented.

*AFD Fovea Detection*. The major characteristic of the AFD is the parabolic appearance of the ILM, again due to deformation by IRF, and, as such, the same method used to compute the fovea position in MFD cases is applied. This is illustrated in Figure 6(a). Furthermore, as with the NFD case a similar correlation was found between the manually annotated fovea positions in AFD cases within the test dataset and distance between ILM and IRF boundary.

### 2.4. Statistical Analysis

The performance of the developed algorithms was evaluated on the unseen validation dataset. For categorical variables (i.e., fovea appearance type), the accuracy was descriptively analysed using confusion matrices and the area under the receiver operating characteristics (ROC) curve. Furthermore, the agreement between the automated and manual diagnosis was evaluated using Pearson correlation. For continuous variables (i.e., fovea position), the distance between manual and automated fovea positions was described as mean with 95% confidence intervals. Furthermore, the correspondence between manual and automated fovea positions was characterized using Pearson's correlation coefficient. The formal significance level was set at *P* < 0.05.

## 3. Results

Implementation of the proposed method was carried out using MATLAB (Version R2012b, The Mathworks Inc.) on an Intel Core i7, 3.5 GHz, with 32 GB RAM.

Of the testing dataset described in Section 2.1, four central RVO (cRVO) and 6 branch RVO (bRVO) scans were excluded due to poor signal and image quality from acquisition. Thus, the validation dataset comprised 230 SD-OCT scans (76 cRVO, 74 bRVO, and 80 nAMD) SD-OCT scans.

### 3.1. Fovea Appearance Classification

Table 1 presents the fovea appearance classification validation, comparing system results with ground truth fovea appearances labelled as either NFD, MFD, or AFD. Automated fovea appearance classification resulted in an 84%, 89%, and 88% correct appearance distinction for bRVO, cRVO, and nAMD, respectively, based on comparison with expert annotation. The area under the ROC curve (AUROC) was computed as 0.956, 0.949, and 0.938 for bRVO, cRVO, and nAMD, respectively. Furthermore, agreement between grader and automated appearance classification using the Cohen's kappa coefficient (*κ*) was *κ* = 0.741, *κ* = 0.804, and *κ* = 0.777 for bRVO, cRVO, and nAMD, respectively.

### 3.2. Fovea Position Detection

Validation of fovea position detection is presented in Table 2 for bRVO, cRVO, and nAMD, showing overall *X*, *Y*, and absolute distance and per device, representing the distances between system results and expert annotated manual fovea positions. For bRVO, *X* fovea position showed a mean ± standard deviation (SD) difference in *μ*m of 92.62 ± 16.48 and 95% confidence interval (CI) of 88.87 to 96.37 and *Y* fovea position showed a mean ± SD difference in *μ*m of 129.1 ± 10.64 and 95% CI of 126.7 to 131.5. For cRVO, *X* fovea position showed a mean ± SD difference in *μ*m of 130.6 ± 61.4 and 95% CI of 116.8 to 144.4 and *Y* fovea position demonstrated a mean ± SD difference in *μ*m of 125.3 ± 31.45 and 95% CI of 118.2 to 132.4. For nAMD, and *X* fovea position showed a mean difference in *μ*m of 160.1 ± 50.09 and 95% CI of 149.1 to 171.1 and *Y* fovea position showed a mean difference in *μ*m of 146.4 ± 43.66 and 95% CI of 136.8 to 155.9.

Correlation between automated and manual fovea positions was tested using the Pearson correlation coefficient. For bRVO, *X* fovea position presents Pearson's *r* = 0.9319 (*P* < 0.0001), and *Y* fovea position *r* = 0.9931 (*P* < 0.0001). For cRVO, *X* fovea position presents Pearson's *r* = 0.9781 (*P* < 0.0001), and *Y* fovea position *r* = 0.9962 (*P* < 0.0001). For nAMD, *X* fovea position presents Pearson's *r* = 0.3493 (*P* < 0.0001) and *Y* fovea position *r* = 0.9717 (*P* < 0.0001).

## 4. Discussion

In this article, we present a fully automated system for classification of the foveal shape and detection of the foveal position in SD-OCT volume scans with exudative maculopathy. Validation against manual ground truth provided by an experienced reading center demonstrated excellent accuracy of the automated system. Furthermore, our method showed applicability across nAMD and RVO diseases as well as across several prevalent SD-OCT devices.

Examination of the results of the first major contribution of this work, fovea appearance classification, shows a correct classification of 84%, 89%, and 88% for bRVO, cRVO, and nAMD cases, respectively, against manually annotated fovea positions. This is further corroborated by receiver operating characteristic where the AUROC was 0.956, 0.949, and 0.938 for bRVO, cRVO, and nAMD, respectively. Thus our method shows a high degree of accuracy for fovea appearance classification when validated using a dataset comprised of variable anatomical fovea appearance (bRVO: NFD = 19, MFD = 4, AFD = 29; cRVO: NFD = 17, MFD = 14, AFD = 39; nAMD: NFD = 53, MFD = 10, AFD = 7 scans).

Analysis of failure cases for bRVO and cRVO scans attribute incorrect appearance classification to incorrect delineation of the ILM surface, as a result of image/signal quality. As can be seen in Table 2, the largest proportion of error cases was MFD classified as AFD in both RVO types. Examination of these cases show poor signal and image quality in the B-scan plane within the retina as a result of acquisition/scanning artefacts and inability for either the layer segmentation described in [21] or our proprietary ILM segmentation to delineate an accurate ILM surface. As a result, the delineated ILM surface is not representative of the actual surface appearance, causing the incorrect classification of MFD as AFD. However, in nAMD cases, the majority of failures occurred when the system identified MFD as NFD. This is attributed to zero thickness computation by the system whereas a human grader has identified an elevation of the foveal depression as dictated by the MFD appearance guidelines.

In the second major contribution of this work, examination of the results of fovea position detection indicates a low absolute distance between the automatically computed fovea positions and manual ground truth for bRVO, cRVO, and nAMD, as well as on a per device basis. Overall, the most accurate automated fovea position detection was seen in bRVO, followed by cRVO, and then nAMD. Examination on a per device basis shows the fovea to be detected most accurately in Spectralis images for bRVO and Cirrus images in both cRVO and nAMD. Based on image quality, it would be expected that detecting the fovea within Spectralis images results in the highest accuracy; however, fovea appearance classification and position detection also rely heavily on the delineation of pathology, in this case, IRFs. As a result, the quality of the imaged pathology, which varies from acquisition to acquisition and is affected by other factors such as imaging artefacts and patient motion, will also play a role in the accuracy of the resulting detected fovea position. Comparisons of the automatically detected fovea positions against their manually annotated counterparts show high correlation (>0.9) for *x* and *y* fovea positions in all disease types, except for *x* fovea position in nAMD. The probable cause for this poorer correlation is likely due to the more variable disease features of AMD in comparison to RVO, thus resulting in more variable *x* fovea position as detected by the automated method. Nevertheless, relative distances between the automatically detected fovea positions and their manually annotated ground truth counterparts remain low.

The examination of automatically detected fovea positions use manually annotated positions for evaluation; thus the accuracy of the ground truth must also be taken into consideration when evaluating the automated results. Not only is this task time consuming to perform precisely for human graders, particularly in a “big data” setting, but also the criteria used to determine the position will also be affected by human subjectivity. Previous studies revealed an mean interobserver variability of 71.63 *μ*m for the foveal position but also a mean distance between the true and device center point of 290.9 *μ*m [15]. Thus, the agreement between human experts is higher compared to our automated system; however, our system accuracy is greater than that obtained by the devices specified in [15], at a mean of 195.5 *μ*m, illustrating the applicability of our method in comparison to other center point detection algorithms and as a more practical alternative to manual center point plotting in a “big data” environment. Furthermore, B-scan spacing must also be considered as the disparity in the number of B-scans from Spectralis, to Cirrus and Topcon varies up to a factor of 1 : 5. As a result, computation of *Y* distance between system and ground truth will be affected. For example, a fovea position misaligned in *Y* dimension by a single B-scan in Cirrus (200 B-scans) would result in an error of ~30 *μ*m, whereas the same misalignment in Spectralis would result in an error of ~125 *μ*m. Thus, such disparity will affect human grader ground truth, and, by extension, system result validation, and given that the test dataset used here features Heidelberg Spectralis scans in the majority (~60%, 143 of 240), explaining the higher distance error reported by the system presented here.

To the best of our knowledge, this is the first fully automated method to locate the position of the fovea directly within retinal SD-OCT scans, independent of whether they are highly pathological due to the presence of cystoid macular edema secondary to central and branch RVO, and neovascular AMD, or within the healthy retina [7] or only via a fundus photograph [8–13]. Future work would concentrate on extension of the IRF segmentation to incorporate a machine learning approach that distinguishes IRF from SRF, allowing for targeted IRF delineation as a feature for computing the fovea position. In addition, the relationship between the fovea region and the thickness of other retinal layers may be explored and used as further anatomical features for detection.

In summary, we have presented a fully automated approach to detect the fovea within healthy and diseased SD-OCT scans of the macula. This enables the use of the fovea as a key landmark in the construction of a population reference frame to identify and extract key spatiotemporal features from a large patient population comprised of different time-points, devices, and imaging modalities. Furthermore, being the functional center of vision, the fovea is crucial for performing analyses of retinal structure/function correlation [22, 23].

## Figures and Tables

**Table 1:** Result of fovea appearance validation, comparing system results with manual expert ground truth fovea appearance classification. The values in bold are scans where the automated fovea appearance classification has identified the fovea appearance correctly based on manual expert ground truth comparison.

		Ground truth											
		bRVO				cRVO				nAMD			
		NFD	MFD	AFD	Total	NFD	MFD	AFD	Total	NFD	MFD	AFD	Total
System	NFD	**30**	2	5	37	**21**	1	5	27	**53**	8	0	61
	MFD	0	**6**	3	9	0	**14**	1	15	2	**10**	0	12
	AFD	2	0	**26**	28	1	0	**33**	34	0	0	**7**	7

	Total	32	8	34	74	22	15	39	76	55	18	7	80

**Table 2:** Results of fovea position validation against expert grader ground truth positions for *X*, *Y*, and absolute distances. Examined are mean distance overall and device specific distances. The lowest distances are highlighted in bold.

	bRVO (*µ*m)			cRVO (*µ*m)			nAMD (*µ*m)		
	*X* (± SD)	*Y* (± SD)	Abs (± SD)	*X* (± SD)	*Y* (± SD)	Abs (± SD)	*X* (± SD)	*Y* (± SD)	Abs (± SD)
Overall	99.48 (78.09)	124.7 (100.2)	159.5 (127.0)	102.0 (85.28)	129.7 (115.7)	165.0 (143.8)	173.1 (225.1)	157.2 (180.4)	262 (262.9)
Spectralis	102.6 (82.56)	**116.5 (96.85)**	**155.2 (127.3)**	**98.78 (80.17)**	150.9 (124.2)	180.4 (147.9)	**120.3 (112.9)**	158.6 (116.6)	199.0 (162.3)
Cirrus	100.4 (73.92)	134.5 (109.3)	167.9 (131.9)	99.02 (97.05)	**79.92 (77.53)**	**127.2 (124.2)**	121.4 (96.16)	**83.94 (96.72)**	**147.6 (136.4)**
Topcon	**67.98 (67.53)**	140.6 (87.70)	156.2 (110.6)	222.7 (45.64)	140.6 (167.5)	263.4 (173.6)	225.5 (291.3)	185.8 (225.6)	325.3 (339.1)
